# Peroxisomal Proliferator-Activated Receptor *β*/*δ* Deficiency Induces Cognitive Alterations

**DOI:** 10.3389/fphar.2022.902047

**Published:** 2022-07-11

**Authors:** Triana Espinosa-Jiménez, Oriol Busquets, Amanda Cano, Elena Sánchez-López, Ester Verdaguer, Antoni Parcerisas, Jordi Olloquequi, Carme Auladell, Jaume Folch, Walter Wahli, Manuel Vázquez-Carrera, Antoni Camins, Miren Ettcheto

**Affiliations:** ^1^ Department of Pharmacology, Toxicology and Therapeutic Chemistry, Faculty of Pharmacy and Food Sciences, University of Barcelona, Barcelona, Spain; ^2^ Biomedical Research Networking Centre in Neurodegenerative Diseases (CIBERNED), Madrid, Spain; ^3^ Institute of Neuroscience, University of Barcelona, Barcelona, Spain; ^4^ Dominick P. Purpura Department of Neurosciences, Albert Einstein College of Medicine, New York City, NY, United States; ^5^ Institute of Nanoscience and Nanotechnology (IN2UB), University of Barcelona, Barcelona, Spain; ^6^ Department of Pharmacy, Pharmaceutical Technology and Physical Chemistry, Faculty of Pharmacy and Food Science, University of Barcelona, Barcelona, Spain; ^7^ Research Center and Memory Clinic, Fundació ACE Institut Català de Neurociències Aplicades—International University of Catalunya (UIC), Barcelona, Spain; ^8^ Unit of Synthesis and Biomedical Applications of Peptides, IQAC-CSIC, Barcelona, Spain; ^9^ Department of Cellular Biology, Physiology and Immunology, Faculty of Biology, University of Barcelona, Barcelona, Spain; ^10^ Departament of Basic Sciences, Universitat Internacional de Catalunya (UIC), Sant Cugat del Vallès, Spain; ^11^ Department of Biochemistry and Physiology, Faculty of Pharmacy and Food Science, University of Barcelona, Barcelona, Spain; ^12^ Department of Biochemistry and Biotechnology, Faculty of Medicine and Life Science, University Rovira i Virgili, Reus, Spain; ^13^ Center for Integrative Genomics, University of Lausanne, Lausanne, Switzerland; ^14^ Lee Kong Chian School of Medicine, Nanyang Technological University, Singapore, Singapore; ^15^ ToxAlim (Research Center in Food Toxicology), INRAE, Toulouse Cedex, France; ^16^ Institute of Biomedicine of the University of Barcelona (IBUB), University of Barcelona, Barcelona, Spain; ^17^ Spanish Biomedical Research Center in Diabetes and Associated Metabolic Diseases (CIBERDEM)-Instituto de Salud Carlos III, Madrid, Spain

**Keywords:** PPARβ/δ, memory impairment, high-fat diet, neuroinflammation, dendritic spines, synapsis, T2DM, insulin signaling

## Abstract

Peroxisome proliferator-activated receptor *β*/*δ* (PPARβ/δ), the most PPAR abundant isotype in the central nervous system, is involved in microglial homeostasis and metabolism, whose disturbances have been demonstrated to play a key role in memory impairment. Although PPARβ/δ function is well-established in metabolism, its contribution to neuronal and specifically memory process is underexplored. Therefore, the aim of the study is to determine the role of PPARβ/δ in the neuropathological pathways involved in memory impairment and as to whether a risk factor implicated in memory loss such as obesity modulates neuropathological markers. To carry out this study, 6-month-old total knock-out for the *Ppard* gene male mice with C57BL/6X129/SV background (PPARβ/δ^-/-^) and wild-type (WT) littermates with the same genetic background were used. Animals were fed, after the weaning (at 21 days old), and throughout their growth, either conventional chow (CT) or a palmitic acid-enriched diet (HFD). Thus, four groups were defined: WT CT, WT HFD, PPARβ/δ^-/-^ CT, and PPARβ/δ^-/-^ HFD. Before sacrifice, novel object recognition test (NORT) and glucose and insulin tolerance tests were performed. After that, animals were sacrificed by intracardiac perfusion or cervical dislocation. Different techniques, such as GolgiStain kit or immunofluorescence, were used to evaluate the role of PPARβ/δ in memory dysfunction. Our results showed a decrease in dendritic spine density and synaptic markers in PPARβ/δ^-/-^ mice, which were corroborated in the NORT. Likewise, our study demonstrated that the lack of PPARβ/δ receptor enhances gliosis in the hippocampus, contributing to astrocyte and microglial activation and to the increase in neuroinflammatory biomarkers. Additionally, alterations in the hippocampal insulin receptor pathway were found. Interestingly, while some of the disturbances caused by the lack of PPARβ/δ were not affected by feeding the HFD, others were exacerbated or required the combination of both factors. Taken together, the loss of PPARβ/δ^-/-^ affects neuronal and synaptic structure, contributing to memory dysfunction, and they also present this receptor as a possible new target for the treatment of memory impairment.

## Introduction

Peroxisome proliferator-activated receptors (PPARs) are ligand-activated transcription factors that belong to the nuclear receptor superfamily (Auwerx et al., 1999). PPARs are activated by natural ligands derived from dietary lipids, such as polyunsaturated fatty acids and their derivatives and exert an important physiological role in regulating glucose, lipid, and lipoprotein metabolism. Likewise, these receptors can be also activated by synthetic ligands like fibrates, glitazones, or nonsteroidal anti-inflammatory drugs (NSAIDs) (Carvajal et al., 2007; Chek Kun et al., 2017), which make them promising targets for several pathologies. Thus, the interest in the medical field for these drug targets has increased exponentially in the last years.

The PPAR subfamily comprises three isotypes: PPARα, PPARγ, and PPARβ/δ. Several studies have shown that PPARα and PPARγ activation mediates by promoting the regulation of pathologic processes including neuroinflammation, mitochondrial alterations, and memory impairment (Heneka et al., 2005; Nicolakakis et al., 2008). Interestingly, although PPARβ/δ has been shown to be the most abundant isotype in the central nervous system (CNS) (Moreno et al., 2004), being expressed in the main cellular components of this system including astrocytes, neurons and microglia (Schnegg and Robbins, 2011), its role in neurodegenerative disorders has not been well characterized.

Inflammation not only actively contributes to the development of several neurodegenerative diseases including Alzheimer´s disease (AD) (Calsolaro and Edison, 2016; Regen et al., 2017), but also plays an essential role in the progression of metabolic pathologies, being a key point where both pathologies converge. In fact, there is multiple evidence that insulin resistance is one of the best predictors of memory impairment supporting the hypothesis that AD represents a form of diabetes mellitus that selectively affects the brain, receiving the name of “type 3 diabetes” (Monte and Wands, 2008). Moreover, several studies demonstrated that people suffering from type 2 diabetes mellitus (T2DM) also develop cognitive decline, which is defined as reduction in one or more cognitive abilities, such as memory (Biessels et al., 2008; Zilliox et al., 2016). In this line, it is well known that obesity actively contributes to the development of T2DM, but also has been related to inflammatory processes and it is considered a clear risk factor in AD (Pugazhenthi et al., 2017; Tumminia et al., 2018; Ebrahimpour et al., 2020). Notably, it has been shown that PPARβ/δ downregulation could be linked to both neuroinflammation and insulin resistance in the brain (De La Monte and Wands, 2006). In fact, several clinical trials have suggested that PPARβ/δ activation reduces inflammation and ameliorates insulin sensitivity (Neels and Grimaldi, 2014; Giordano Attianese and Desvergne, 2015; Vázquez-Carrera, 2016), among others. Therefore, they have been considered as good candidates for the treatment of these pathologies characterized by these hallmarks, such as T2DM (Salvadó et al., 2012). In the CNS, synthetic PPARβ/δ-specific agonists have been reported to ameliorate clinical symptoms, reducing the severity of a variety of CNS pathologies by modulating oxidative stress and inflammatory responses associated with these diseases (Tong and Dominguez, 2016; Tong et al., 2016).

Collectively, although PPARβ/δ seems to play a key role in several pathologic processes, including memory impairment, the mechanisms responsible for these effects remain unknown. Therefore, the aim of the study is to determine the role of PPARβ/δ in the neuropathological pathways involved in the development of memory impairment and as to whether a risk factor involved in cognitive loss and in the development of T2DM such as obesity (high-fat diet, HFD) consumption) modulates hippocampal neuropathological markers in mice lacking this nuclear receptor.

## Materials and Methods

### Animals

To perform this study, 6-month-old total knock-out for the PPARβ/δ gene male mice with C57BL/6X129/SV background (PPARβ/δ^-/-^) (Karim et al., 2006) and wild-type (WT) littermates with the same genetic background were used. In all cases, animals were obtained from established breeding couples in the animal facility (Animal facility from the Pharmacy and Food Sciences Faculty from the University of Barcelona; approval number C-0032). After the weaning (at 21 days-old), and throughout their growth, animals were fed either conventional chow (control diet, CT) (ENVIGO, Madison, Wt 53744-4220) or a palmitic acid-enriched diet containing 45% of fat mainly from hydrogenated coconut oil (HFD) (Research Diets Inc., NB, United States). Thus, four groups were defined: WT CT, WT HFD, PPARβ/δ^-/-^ CT, and PPARβ/δ^-/-^ HFD.

All animals were kept under stable conditions of humidity and temperature, standard light-dark cycle (12-h light/dark cycle) and food and water *ad libitum*, following the ethics guidelines defined by the European Committee (European Communities Council Directive 2010/63/EU). Manipulation protocols were previously approved by the ethics committee from the University of Barcelona and, at all times, it was made sure that animal numbers, their stress, and pain were kept under a necessary minimum following the appropriate animal manipulation ethical methodologies.

### Glucose and Insulin Tolerance Tests

For both tests, mice were fasted for 6 h and the tests were performed in a quiet room, preheated to +28°C. In the glucose tolerance test (GTT), glucose was administered at a dose of 1 g/kg intraperitoneally (i.p). On the other hand, in the insulin tolerance test (ITT), the dosage of insulin used was 0.75 IU/Kg and it was also administered i.p. Next, samples from the tail vein were extracted in consecutive periods of time and glucose levels were measured using an Accu-check®Aviva glucometer. In GTT, measurements were made at 5, 15, 30, 60, and 120 min after the administration of glucose. In the ITT case, samples were extracted at 15, 30, 45, 60, and 90 min after the insulin administration.

The animals were monitored in every moment, and in those cases where glucose levels dropped under a concentration of 20 mg/dl, a dosage of 1 g/Kg of glucose was administered i.p. and they were kept in observation until blood glucose concentrations were stable and the animal behavior was normal. Twelve animals per group were analyzed.

### Novel Object Recognition Test

This behavioral test is used for testing the hippocampal-dependent recognition memory of mice based on the spontaneous tendency of rodents to spend more time exploring a novel object than a familiar one. It consists of three phases: habituation, familiarization, and test phase. In the first one, mice were placed in a circular open-field arena of 40 cm in diameter surrounded by black curtains where the light intensity in the middle of the field was 30 lux. Their tracking was monitored (Smart 3.0; Panlab) for three consecutive days for 10 min each mouse. In the fourth day, two identical objects (A-A’) were placed in the arena at an equal distance and 24 h later, one object was replaced by a new one (A-B) and the exploration time (10 min) of each mouse was measured. Exploration was defined as the orientation of snout of the animals toward the object, sniffing or touching (Antunes and Biala, 2012; Ettcheto et al., 2016). In those cases, when the total time exploring both objects was less than 5 min, the mouse was excluded. Data were measured by discrimination index (DI), which indicates the difference in exploration time in seconds between familiar and novel object, using the followingt equation:

$$DI=\frac{\mathrm{B exploration time}-\mathrm{A exploration time}}{\mathrm{Total exploration time}}$$

All spaces were properly cleaned with 96% ethanol between animals, in order to eliminate odor or other cues.

### Hippocampal Spine Density Analysis

To carry out the spine density analysis, five animals per group were used which were sacrificed by cervical dislocation. After, the brain was isolated, it was processed following the instructions of the GolgiStainTM Kit purchased from FD Neurotechnologies, Inc. (FD Rapid GolgiStainTM Kit; Cat ^#^PK401). Images were obtained with a BX61 Laboratory Microscope (Melville NY-Olympus America Inc.). The quantification was carried out by selecting five neurons per animal in the dentate gyrus (DG) of the hippocampus. Measurement was done at least 50 μm from the soma along consecutive 10 μm on secondary branches starting 10 μm after branching from the primary dendrite. Spine density was calculated by dividing the number of spines per segment by the length of the segment and was expressed as the number of spines per 10 μm of dendrite.

### Immunoblot Blot Analysis

Fresh brains of at least four mice per group were extracted right after euthanasia (cervical dislocation) and the hippocampus were dissected and kept frozen at −80°C until use. Next, samples were homogenized in lysis buffer (Tris HCl 1 M pH 7.4, NaCl 5 M, EDTA 0.5 M pH 8, Triton, distilled H20) containing protease and phosphatase inhibitor cocktails (Complete Mini, EDTA-free; Protease Inhibitor cocktail tablets, 11836170001, Roche Diagnostics GmbH, Germany; Phosphatase Inhibitor Cocktail 3, P0044, Sigma-Aldrich, United States). The samples were centrifuged at 14,000 rpm for 10 min at 4°C after a 30-min incubation at the same temperature. The supernatant was recovered and frozen at −80°C until use.

Sample protein concentration was determined using the PierceTM BCA Protein Assay Kit (Thermo ScientificTM). For immunoblot assays, 10 µg per sample was used and denatured at 95°C for 5-min in a sample buffer [0.5 M Tris HCl, pH 6.8, 10% glycerol, 2% (w/v) SDS, 5% (v/v) 2-mercaptoethanol, 0.05% bromophenol blue]. Electrophoresis was performed on acrylamide gels of 7%, 10%, and 12% concentration at constant 120 V and transferred to polyvinylidene difluoride sheets (Immobilon®-P Transfer Membrane; IPVH00010; Merk Millipore Ltd., United States) at constant 200 mA for 120 min. Then, membranes were blocked for 1-h with 5% non-fat milk dissolved in TBS-T buffer [0.5 mM Tris; NaCl, Tween® 20 (P1379, Sigma-Aldrich, United States), pH 7.5], washed with TBS-T 3 times for 5-min, and incubated with the appropriate primary antibody, detailed in Supplementary Table S1, overnight (O/N) at 4°C. Subsequently, blots were washed in TBS-T buffer and incubated at room temperature for 1 h with the appropriate secondary antibody (Supplementary Table S1). Finally, results were obtained through chemiluminescence detection using the Pierce® ECL Western Blotting Substrate (^#^32106, Thermo Scientific, United States), a Bio-Rad Universal Hood II Molecular Imager, and the Image Lab v5.2.1 software (Bio-Rad laboratories). Measurements were expressed in arbitrary units and all results were normalized with the corresponding loading control (Glyceraldehyde-3-phosphate dehydrogenase; GAPDH).

### Immunofluorescence

At least four animals per group were previously anesthetized by the i.p. injection of ketamine (100 mg/Kg) and xylazine (10 mg/Kg). When they were in the no-pain sleep phase, they were intracardially perfused with 4% paraformaldehyde (PFA) diluted in 0.1 M phosphate buffer (PB). After perfusion, brains were removed and stored in 4% PFA O/N at 4°C. The next day, the solution was changed into 4% PFA+30% sucrose. Coronal sections of 20 µm were obtained by a cryostat (Leica Microsystems) and kept in a cryoprotectant solution at −20°C until their use.

On the first day of the assay, free-floating sections were washed three times with 0.1 M PBS pH 7.35 and after, five times with PBS-T (0.1 M PBS; 0.2% Triton X-100). Then, they were blocked in a solution containing 10% fetal bovine serum (FBS) and 1% Triton X-100 diluted with PBS-T five times for 5 min each and incubated with the primary antibody (Supplementary Table S2) O/N. On the second day, slices were washed with PBS-T 5 times for 5 min and incubated with the pertinent secondary antibody (Supplementary Table S2) for 2 h at room temperature. Finally, sections were treated with 0.1 μg/ml Hoechst (Sigma-Aldrich, St Louis, MO, United States), used for cell nuclei staining, for 8 min in the dark at room temperature and washed with 0.1 M PBS. All reagents, containers and materials exposed to Hoechst were properly managed and processed to avoid any cytotoxic contamination. Finally, brain slices were mounted in gelatin-coated slides using Fluoromount G (EMS) and were left to dry O/N. Image acquisition of dentate gyrus of hippocampus was obtained in a blinded manner using an epifluorescence microscope (BX61 Laboratory Microscope, Melville, NY-Olympus America Inc.) and quantified by ImageJ.

### Statistical Analysis

All results are presented as MEAN ± SD. Groups were compared against each other using two-way ANOVA. When variables independently were significant, we denoted ^#^ *p* < 0.05, ^##^ *p* < 0.01, ^###^ *p* < 0.001, and ^####^ *p* < 0.0001 for the diet factor and ^$^ *p* < 0.05, ^$$^ *p* < 0.01, ^$$$^ *p* < 0.001, and ^$$$$^ *p* < 0.0001 for the genotype factor. When the interaction between two analyzed factors in ANOVA was significant, Tukey’s post hoc test was performed for comparison among groups (∗*p* < 0.05. ∗∗*p* < 0.01, ∗∗∗*p* < 0.001, and ∗∗∗∗*p* < 0.0001). All analyses and GAPDH representations were obtained using Graph Pad Prism software for Mac version 6.01; Graph Pad Software, Inc.

## Results

### HFD Feeding Induces Body Weight Increase and Glucose Alterations at Peripheral Level

**FIGURE 1:**
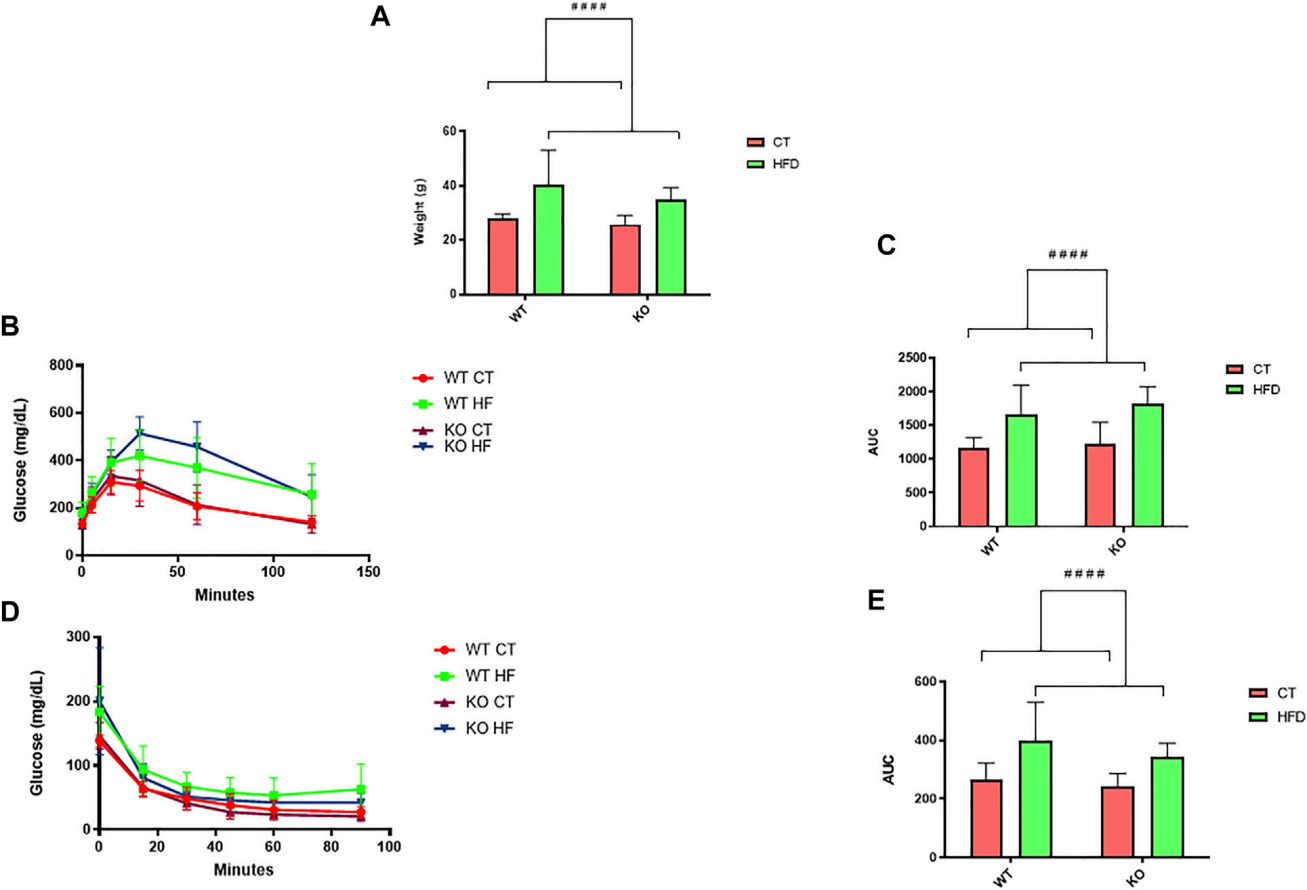
**(A)** Analysis and representation of changes in body weight (*n* = 12 animals per group). **(B)** GTT and **(D)** ITT experiment profiles (*n* = 12 animals per group). Area under curve (AUC) data were calculated from the time point 0 until the end of the experiment for both **(C)** GTT and **(E)** ITT. Statistical analysis was performed through two-way ANOVA. Significant differences were found between control and high-fat diet groups: ^####^ denote *p* < 0.0001.

Body weight profile was analyzed at 6 months of age in WT and PPARβ/δ^-/-^ mice after being fed conventional chow or HFD from their weaning. Two-way ANOVA revealed that feeding HFD significantly increased (*p* < 0.0001) their body weight in both genotypes compared to mice fed the control diet (Figure 1A), reaching an increase of 135% in body weight in HFD vs. CT. Moreover, GTT and ITT were performed in order to evaluate peripheral alterations of glucose metabolism in these experimental groups. In line with our previous studies, two-way ANOVA showed a significant effect of diet variable in both GTT (*p* < 0.0001) and ITT (*p* < 0.0001), with an increase of 145% due to HFD feeding in both tests, thereby indicating that feeding the HFD affects both genotypes (Figures 1B–E).

### Evaluation of Memory Impairment

**FIGURE 2:**
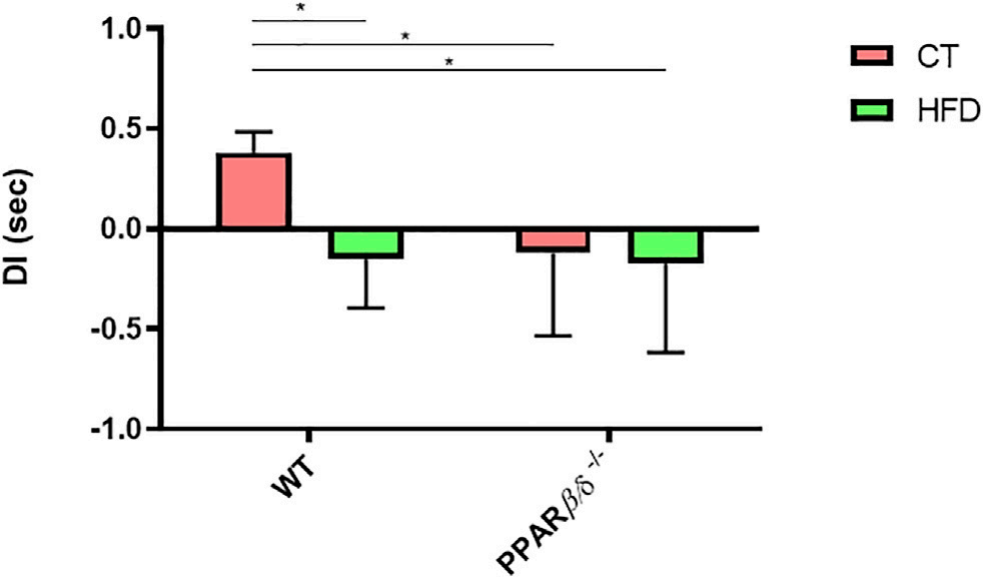
NORT, DI expressed in seconds (Number of animals ≥10). Statistical analysis was performed by two-way ANOVA and Tukey post-test (WT CT vs. WT HFD: * denotes *p* < 0.05; WT CT vs. PPAR β/δ^-/-^ CT: * denotes *p* < 0.05; WT CT vs. PPAR β/δ^-/-^ HFD * denotes *p* < 0.05).

To determine the impact of PPARβ/δ deficiency on memory function together with HFD intake, we evaluated the hippocampal-dependent recognition memory by Novel Object Recognition Test (NORT) (Figure 2). Two-way ANOVA revealed a significant effect of diet and genotype (*p* < 0.05 and *p* < 0.05, respectively) with interaction of both variables (*p* < 0.05). Therefore, Tukey’s post hoc was performed which showed that HFD feeding promoted a significant reduction of 39% in the memory capacity in WT animals. However, PPAR β/δ^-/-^ mice demonstrated to have a significant decrease in recognition memory compared to WT CT independently of the diet (WT CT vs*.* WT HFD *p* < 0.05; WT CT vs*.* PPAR β/δ^-/-^ CT *p* < 0.05; WT CT vs*.* PPAR β/δ^-/-^ HFD *p* < 0.05).

### PPARβ/δ^-/-^ Mice and Obesity Are Associated With Decreased Dendritic Spine Density

**FIGURE 3:**
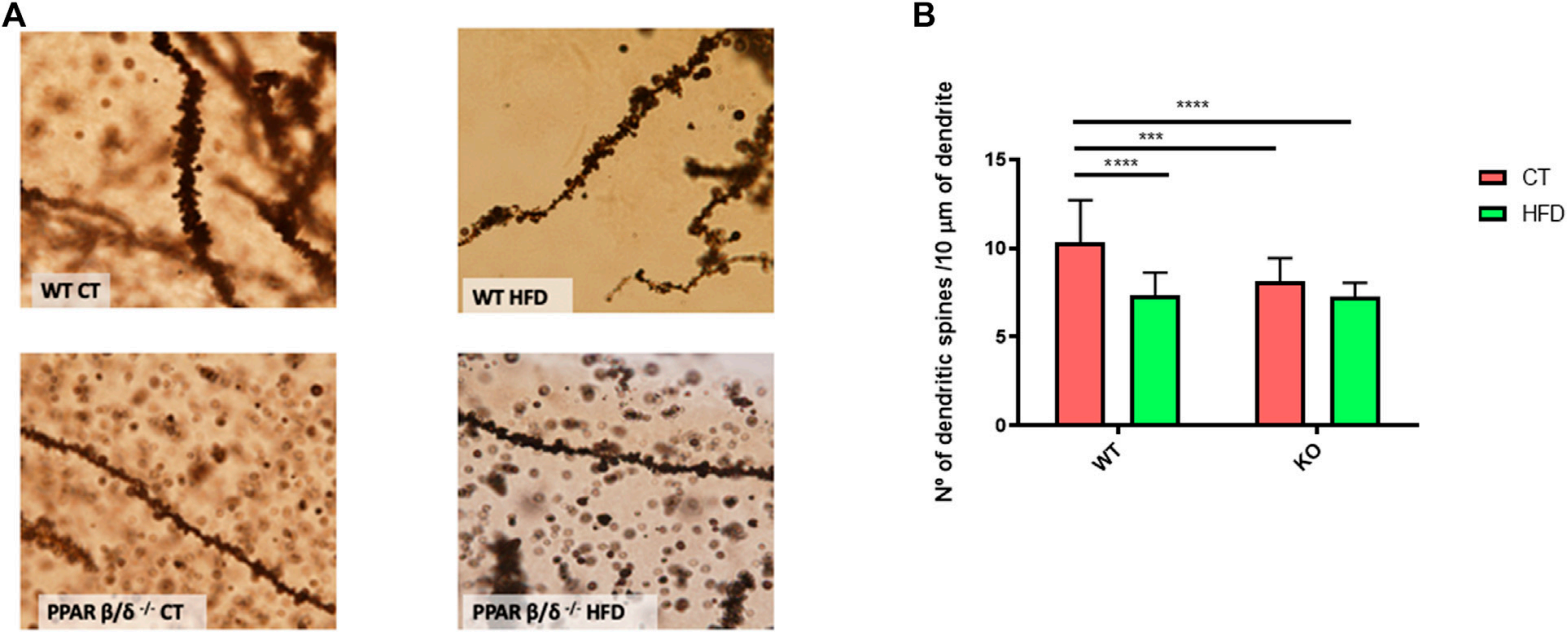
**(A)** Optical microscope images of the DG of hippocampus GolgiStain. Scale bar: 10 μm. **(B)** Quantification of dendritic spines for each 10 μm. Groups were compared against each other using two-way ANOVA and Tukey post-test (*n* = 5) (WT CT vs. WT HFD: **** denote *p* < 0.0001; WT CT vs. PPAR β/δ^-/-^ CT: *** denote *p* < 0.001; WT CT vs. PPAR β/δ^-/-^ HFD: **** denote *p* < 0.0001).

The memory process was also evaluated by the analysis of dendritic spine density in the hippocampus, since it has been demonstrated that the number of dendritic spines positively correlates with synaptic plasticity and cognition (Yuste and Bonhoeffer, 2001; Sala and Menahem, 2014). In our study, the dendritic spine detection and subsequent quantification were performed where the obtained results correlated with those of the behavioral test. Specifically, our data demonstrated a significant effect of diet and genotype (*p* < 0.01 and *p* < 0.0001, respectively) with the interaction between both variables (*p* < 0.01). Following, Tukey´s post hoc was performed and our results showed a significant decrease of 30% in hippocampal spine number in WT mice after being fed with HFD (*p* < 0.0001). By contrast, animals with PPARβ/δ deficiency also exhibited a significant reduction compared to WT, but HFD feeding did not induce a synergic effect (WT CT vs. PPAR β/δ^-/-^ CT 22%, *p* < 0.001; WT CT vs. PPAR β/δ^-/-^ HFD 30%; *p* < 0.0001) (Figures 3A,B). Moreover, as can be observed in Figure 3A, the reduction in spine number was accompanied by shorter and smaller dendritic spines (qualitative evaluation). Therefore, our results indicated that HFD feeding induces memory alterations in WT. However, when animals exhibit PPARβ/δ deficiency memory profile was similar, but it was not enhanced by the diet.

**FIGURE 4:**
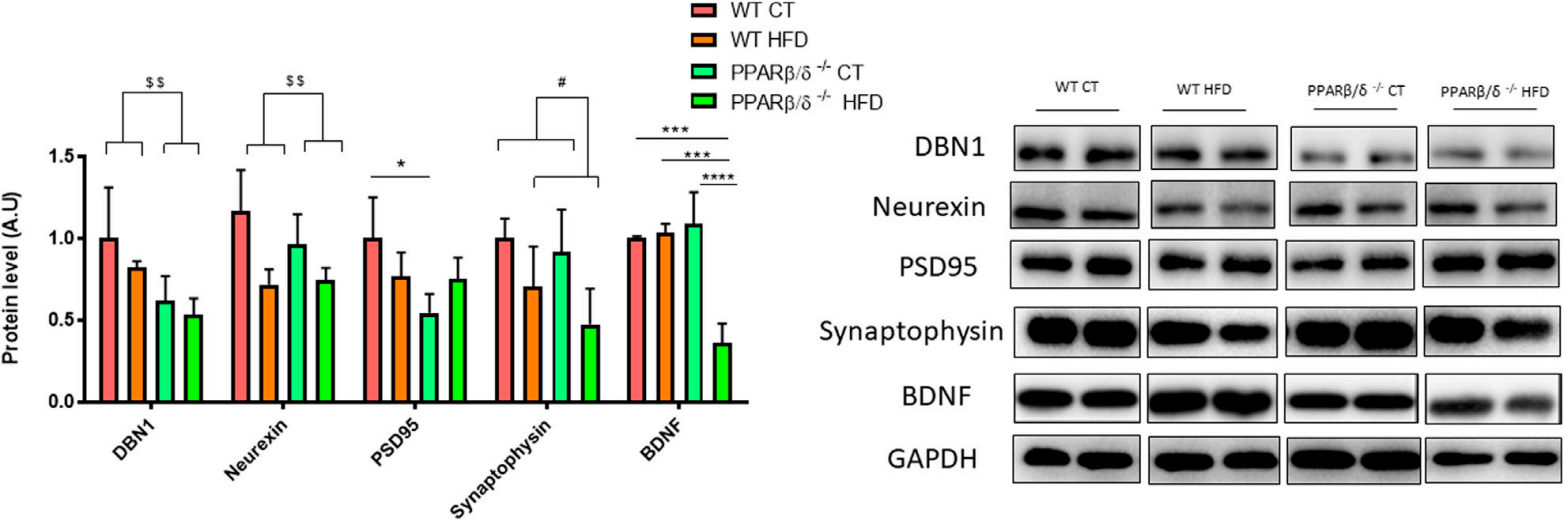
Immunoblot detection of synaptic proteins where two representative samples out of four per group are shown. All results are presented as MEAN ± SD. Data were analyzed by two-way ANOVA (*n* ≥ 4) (DBN1 and neurexin:WT vs. PPAR β/δ^-/-^: ^$$^ denotes *p* < 0.01) (synaptophysin: CT vs. HFD: ^#^ denote *p* < 0.05). In the case of PSD95 and BDNF, Tukey post-test was performed (PSD95: WT CT vs. PPAR β/δ^-/-^ CT: *denotes *p* < 0.05) (BDNF: WT CT vs*.* PPAR β/δ^-/-^ HFD: *** denote *p* < 0.001; WT HFD vs*.* PPAR β/δ^-/-^ HFD: *** denote *p* < 0.001; PPAR β/δ^-/-^ CT vs*.* PPAR β/δ^-/-^ HFD: **** denotes *p* < 0.0001.

In order to support these findings, proteins directly involved in memory process and plasticity in the hippocampus such as the drebrin 1 (DBN1), neurexin, density protein 95 (PSD95), synaptophysin, and brain-derived neurotrophic factor (BDNF) were evaluated. DBN1 has been demonstrated to play a key role in dendritic spine regulation (Ivanov et al., 2009a; Ivanov et al., 2009b). In this line, two-way ANOVA revealed a significant reduction (*p* < 0.01) in DBN1 protein levels in PPARβ/δ^-/-^ mice compared with WT mice. Likewise, neurexin (a presynaptic protein) showed the same pattern, a significant implication of the genotype (*p* < 0.01) in its protein level. Moreover, a significant effect of the genotype (*p* < 0.05) also was observed in PSD95, (a postsynaptic protein) but in this case, with interaction between two variables (*p* < 0.05). Therefore, Tukey’s post hoc was carried out to analyze the differences among the experimental groups. Our results exhibited a significant reduction in PSD95 in PPARβ/δ^-/-^ CT compared to WT CT. Regarding synaptophysin, this protein is expressed in synaptic vesicles and its reduction has been associated with impairments to neuronal health together with BDNF (J. T. Yang et al., 2019). In this line, our results showed a significant reduction of synaptophysin in those animals fed HFD, demonstrating a significant effect of this variable (*p* < 0.05), but this reduction was not exacerbated by the absence of PPAR β/δ. Finally, concerning BDNF, results showed a significant effect of the diet (*p* < 0.001) and genotype (*p* < 0.01) with the interactions between both variables (*p* < 0.001). Next, Tukey´s post hoc test revealed a significant decrease in hippocampal BDNF protein levels in PPARβ/δ^-/-^ mice fed HFD compared to the other groups (WT CT vs. PPAR β/δ^-/-^ HFD *p* < 0.001; WT HFD vs. PPAR β/δ^-/-^ HFD *p* < 0.001; PPAR β/δ^-/-^ CT vs. PPAR β/δ^-/-^ HFD *p* < 0.0001) (Figure 4). Collectively, as previously described (Saiyasit et al., 2020), our results confirm that HFD feeding contributes to disturbances in the synaptic transmission. However, when there is a PPAR β/δ deficiency, biomarkers related to neuronal function are directly altered independently of diet, suggesting the essential role that PPAR β/δ plays in the synaptic transmission.

### PPAR β/δ Deficiency Increases Glial Markers Activation

**FIGURE 5:**
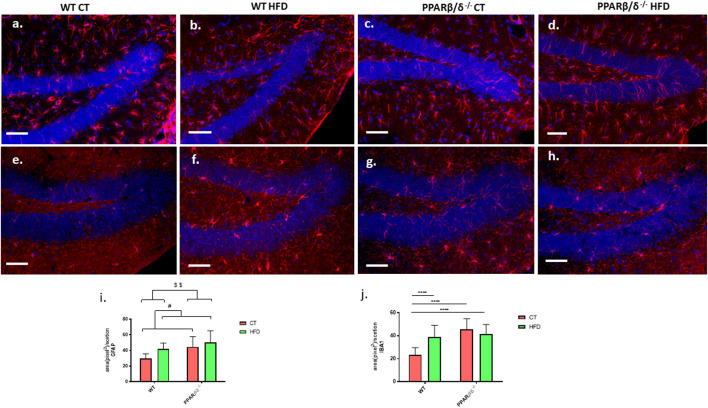
Evaluation of inflammatory responses. Representative images for the detection of astrocytes **(A–D)** and microglia **(E–H)** (red color in both cases) in the DG of the hippocampus. All samples are co-stained with Hoechst for the detection of cellular nucleus (blue). Scale bar: 200 μm. Graphic representation of fluorescence intensity for GFAP **(I)** and IBA1 **(J)**. In the case of GFAP, statistical analysis was performed through two-way ANOVA (Number of animals ≥4) (Control diet vs. HFD: ^#^ denote *p* < 0.05) (WT vs. PPAR β/δ^-/-^: ^$$^ denote *p* < 0.01). For IBA1, two-way ANOVA and Tukey’s were used for statistical analysis (WT CT vs. WT HFD: **** denote *p* < 0.0001, WT CT vs. PPAR β/δ^-/-^ CT: **** denote *p* < 0.0001, WT CT vs. PPAR β/δ^-/-^ HFD: **** denote *p* < 0.0001).

Evaluation of the reactive profile of astrocytes and microglia in the hippocampal dentate gyrus was performed through the detection of glial fibrillary acidic protein (GFAP) and ionized calcium-binding adapter molecule 1 (IBA1) proteins by immunohistofluorescence. Representative images of all four experimental groups can be found in Figure 5. Moreover, graphic quantification of fluorescence intensity measured by ImageJ is shown in Figure 5I for GFAP and j for IBA1. In the case of GFAP, two-way ANOVA showed a significant effect of diet (*p* < 0.05) and genotype variables (*p* < 0.01) without interaction between them, demonstrating that, on the one hand, HFD induced a significant activation of astrocytes compared to control diet, and on the other, PPARβ/δ^-/-^ mice presented a significantly higher basal profile of reactive astrocytes than WT mice (Figure 5I). In the study of microgliosis, our results revealed a significant effect of both diet (*p* < 0.01) and genotype (*p* < 0.0001) with an interaction between both factors (*p* < 0.0001). Subsequently, Tukey’s post hoc showed a significant microgliosis in WT mice fed HFD compared to those fed conventional chow (*p* < 0.0001). In the case of PPAR β/δ^-/-^ mice, both groups exhibit a significant microglial activation compared to WT regardless of the consumed diet (PPARβ/δ^-/-^ CT vs. WT CT *p* < 0.0001; PPARβ/δ^-/-^ HFD vs. WT CT) (Figure 5J).

**FIGURE 6:**
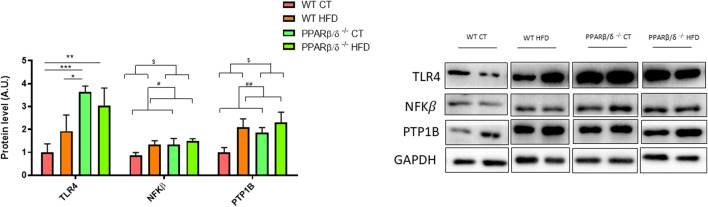
Semi-quantification of protein levels for TLR4, NFKβ, and PTP1B where two representative samples out of four per group are shown. All results are presented as MEAN ± SD. Groups were compared against each other using two-way ANOVA (*n* ≥ 4) (CT vs. HFD: ^##^ denote *p* < 0.01; WT vs. PPAR β/δ^-/-^: ^$^ denotes *p* < 0.05). In the case of TLR4, Tukey post-test was performed (WT CT vs. PPAR β/δ^-/-^ HFD: ** denote *p* < 0.01; WT CT vs. PPAR β/δ^-/-^ CT: *** denote *p* < 0.001; WT HFD vs. PPAR β/δ^-/-^ CT: * denotes *p* < 0.05).

At the molecular level, the levels of different proteins related to the neuroinflammation process were analyzed in the hippocampus including toll-like receptor 4 (TLR4), nuclear factor kappa B(NFKβ), and protein tyrosine phosphatase (PTP1B). Regarding TLR4 protein levels, two-way ANOVA showed a significant effect of genotype (*p* < 0.05) with the interaction between both variables, genotype and diet (*p* < 0.05). Next, Tukey’s post hoc revealed a significant TLR4 increase in PPARβ/δ^-/-^ mice compared to WT fed the standard chow diet (WT CT vs. PPARβ/δ^-/-^ CT *p* < 0.001; WT CT vs. PPARβ/δ^-/-^ HFD *p* < 0.01; WT HFD vs. PPARβ/δ^-/-^ CT *p* < 0.05) (Figure 6).

In the case of NFKβ and PTP1B, two-way ANOVA revealed a significant effect of diet and genotype for both proteins (*p* < 0.05 and *p* < 0.05; *p* < 0.01 and *p* < 0.05, respectively), although none of them demonstrated interaction between both factors (Figure 6). Collectively, our results confirm that feeding the HFD and the lack of PPARβ/δ differently affect the levels of proteins involved in neuroinflammation.

### PPAR β/δ Deficiency Disrupts the Insulin Signaling Pathway in the Hippocampus

**FIGURE 7:**
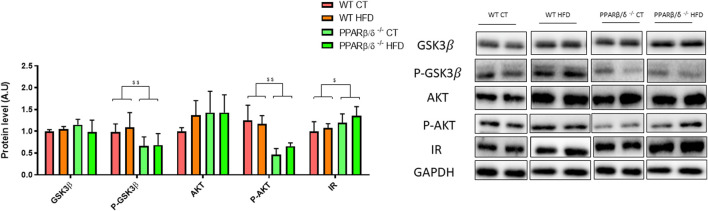
Immunoblot detection of IR and related signaling proteins where two representative samples out of four per group are shown. All results are presented as MEAN ± SD. Groups were compared against each other using two-way ANOVA (*n* ≥ 4) (WT vs. PPAR β/δ^-/-^: ^$$^ denote *p* < 0.01; ^$^ denote *p* < 0.05).

Since previous studies have demonstrated that hippocampal insulin signaling plays a key role in the memory processes (McNay and Recknagel, 2011; Talbot et al., 2012; Barber et al., 2021), we next evaluated different proteins involved in this molecular pathway, including glycogen synthase kinase 3 beta (GSK3β) and protein kinase B (AKT). The phosphorylation levels of these proteins showed a similar profile. When two-way ANOVA was applied, a significant effect of the genotype was observed (*p* < 0.01 and *p* < 0.01) without interaction between genotype and diet, thereby suggesting that PPARβ/δ deficiency reduces their activation. However, GSK3β and AKT did not show a significant effect on variables after statistical analysis. Surprisingly, when insulin receptor (IR) was evaluated, results showed that the genotype significantly affected IR levels (*p* < 0.05), probably to compensate for the inefficacy of the pathway (Figure 7).

## Discussion

The present work provides novel findings on the neurological role of PPARβ/δ in mice under standard conventional chow diet and after HFD intake, which indicate that PPARβ/δ plays a prominent role in dendritic spine preservation and memory process; therefore, it may protect against the memory impairment. Interestingly, HFD consumption does not exacerbate brain cognitive pathology observed in the PPARβ/δ-deficient mice at this level. These results are in agreement with those observed in central insulin signaling and neuroinflammation markers, reinforcing the importance of this receptor at central level. On the other hand, our data demonstrated that alterations in body weight, GTT, and ITT were induced due to continuous HFD intake and not due to the lack of this nuclear receptor, probably due to continued dietary intervention. Thus, using the well-established line of PPARβ/δ^-/-^ mice and its WT controls, we assessed the impact of PPARβ/δ deficiency on cognitive function, synaptic plasticity, dendritic spine density, and synaptic markers, and evaluated whether HFD intake deteriorates this pathological status.

It has been widely demonstrated that the PPAR superfamily plays a key role in metabolic processes. In fact, their agonists are used for the treatment of pathologies including T2DM. Specifically, it has been demonstrated that PPARβ/δ is expressed throughout the brain, with prominent localization in the mouse hippocampus, entorhinal cortex, and hypothalamus (Braissant et al., 1996; Heneka and Landreth, 2007; Hall et al., 2008). Interestingly, several studies have demonstrated the link between metabolic dysfunctions and memory impairment. In fact, AD is considered as type 3 diabetes (Small et al., 2000; Leszek et al., 2017). Therefore, taking all these into account, the interest in this receptor and its agonists has increased for potential therapeutic interventions in the treatment of AD and cognitive disorders related to obesity.

Regarding metabolic alterations, PPAR β/δ agonists substantially decrease adiposity and improve glucose intolerance and insulin resistance in animal models (Barak et al., 2002; Nicolakakis et al., 2008; Salvadó et al., 2012; Tan et al., 2016; Palomer et al., 2018). However, it has been observed that PPAR β/δ-null mice showed a significant body weight reduction compared to control mice in the first week after birth, differences that disappear when they are adults (Peters et al., 2000). These data agree with the results presented in this study in which we did not observe changes in weight depending on the genotype.

On the other hand, it is well known that feeding the HFD causes a significant increase in body weight, glucose intolerance, and insulin resistance, (Musso et al., 2003; Álvarez-Amor et al., 2021). However, we did not find alterations in these parameters due to the genotype. One possible explanation for these discrepancies could be the differences in time exposition to the HFD. In this context, previous studies have exposed animals to a hypercaloric diet following a different pattern compared to ours in which animals were fed from their weaning at 21 days old until their sacrifice at 6 months. Therefore, in line with previous studies, young animals exposed to a HFD might show more plasticity to adapt to the diet (Burke et al., 2021) and for this reason, our mice did not show differences due to lack of the receptor.

It is well known that dendritic spines play a pivotal role in the learning process, whereas synaptic plasticity alterations have been directly correlated to memory impairments (Terry et al., 1991; Scheff et al., 2006; Bourne and Harris, 2007; Knott and Holtmaat, 2008; Chidambaram et al., 2019; Ettcheto et al., 2020). This prompted us to focus our study on the evaluation of these structures in order to assess the effect of PPARβ/δ deficiency in dendritic spines density at the hippocampal level, since PPARβ/δ has been associated with the neuroinflammatory process and insulin resistance in the brain, which are considered important contributors for neurodegeneration. In this line, our results highlighted that PPARβ/δ^-/-^ mice showed a significant reduction of these structures in mice fed a standard diet and this was not exacerbated by the HFD, suggesting that this receptor is necessary for the maintenance of dendritic spines and, in consequence, for eluding memory impairment. By contrast, in WT mice, we observed a reduction of dendritic spines due to HFD intake for a long period of time. These results agreed with previous studies that demonstrated that HFD-induced obesity is considered a clear risk factor for the development of AD. These data were correlated with those obtained in the NORT, also in previous studies performed by our group (Barroso et al., 2013). Moreover, it has been widely demonstrated that PPARβ/δ plays a key role in running endurance (Wang et al., 2004; Schuler et al., 2006; Röckl et al., 2007; Thomson et al., 2007). PPAR β/δ is the most expressed isoform in the skeletal muscle, especially in the oxidative fibers. These fibers mainly expressed enzymes involved in the fatty acids oxidation. In this line, it has been demonstrated that the overexpression of PPARβ/δ in this tissue induces the reorganization of these fibers increasing the percentage of oxidative fibers and decreasing muscle fatigue (Hämäläinen and Pette, 1995), being crucial also for endurance (Wang et al., 2004). Therefore, the lack of this isotype has been associated with alterations in physical performance that imply an intense exercise including swimming or running. Therefore, the lack of PPARβ/δ may affect the swimming performance, making MWM unreliable to assess the mice cognition.

On the other hand, it is known that obesity-associated with HFD intake heightens alterations in dendritic spine density, neuronal loss, and memory impairment through several mechanisms, including neuroinflammation (Stranahan et al., 2011; Duffy et al., 2019). Our results confirm this dendritic spine reduction in WT fed HFD compared to WT mice fed the standard chow.

Going in depth at the molecular level, DBN1 is an actin-binding protein abundant within dendritic spines, which is typically located in postsynaptic receptive regions of excitatory synapses (Sekino et al., 2007) and it is thought that it controls spine morphology and function (Hayashi et al., 1996). In fact, its reduction in the hippocampus has been correlated with cognitive deficits and, by contrast, its preservation has been associated with neuroprotection (Harigaya et al., 1996; Hatanpää et al., 1999; Kojima and Shirao, 2007; Counts et al., 2012). In line with these previous studies, our study showed a significant decrease in DBN1 protein level in the hippocampus induced by PPARβ/δ deficiency. This is in agreement with the findings obtained in dendritic spine analysis. Likewise, it has been reported that *dbn1* loss in the brain is not sufficient to induce synaptic dysfunction (Willmes et al., 2017). Therefore, our study suggests that PPARβ/δ actively contributes to the preservation of these structures together with DBN1. Consistent with this, neurexin and PSD95 protein levels also showed a similar pattern, which confirms that PPARβ/δ plays a pivotal role in synaptic plasticity.

Regarding synaptophysin, the reduction of this glycoprotein, which predominates in the synaptic vesicles, has been related to memory impairment (Zhang et al., 2019; Du et al., 2020) In this context, as it has been previously commented, obesity also plays a key role in the correct function of memory interconnecting these three concepts (Daniels et al., 2005; Kalarchian and Marcus, 2012). Our results showed a significant decrease in synaptophysin protein levels in the hippocampus induced by the HFD feeding. However, we did not observe any significant reduction related to the genotype. The neuronal activity has also been described to regulate BDNF transport into dendrites, which have been involved in the modulation of synaptic transmission and synaptogenesis (Lu and Figurov, 1997; Tongiorgi et al., 1997; Tongiorgi, 2008). In this study, we observed a significant reduction in its levels in PPARβ/δ^-/-^ fed HFD compared to the other groups, suggesting that PPARβ/δ deficiency alone is not enough to alter BDNF protein levels at this time.

Neuroinflammation is a common feature of every central nervous system diseases and is being highly recognized as a potential mediator of memory impairment (Fourrier et al., 2019; McGrattan et al., 2019). The impact of this complex process, which includes the alteration of the TLR4 pathway and glial activation among other processes, induces the release of pro-inflammatory cytokines and aberrant neuronal circuits, contributing altogether to the acceleration of cognitive decline (Zhang et al., 2018a; Jin et al., 2019; Malpetti et al., 2020). However, the involvement of inflammation is not fenced only in the brain. In fact, it has been demonstrated that HFD intake and obesity also impair cognitive function in animal models (Kanoski and Davidson, 2011), as well as in humans (Power et al., 2015), by disrupting hippocampal morphology and synaptic plasticity caused by inflammation (Porter et al., 2010; Wang et al., 2015). In this context, PPARβ/δ has been involved in the modulation of inflammation at both peripheral and central levels. Our study demonstrated that the lack of PPARβ/δ receptor enhances the gliosis in the hippocampus, contributing to astrocyte and microglial activation. Likewise, TLR4 and NFKβ protein levels also showed a significant increase in PPAR β/δ^-/-^ mice. In agreement with these data, studies performed by Rodríguez-Calvo and colleagues demonstrated that the PPARβ*/*δ agonist GW501516 inhibited LPS-induced cytokine production by preventing NFKβ activation (Rodríguez-Calvo et al., 2008). Interestingly, HFD promoted similar alterations to those observed when there is a deficiency of PPARβ*/*δ. Exceptionally, in the case of microglial activation, whereas feeding HFD induced neuroinflammation in WT mice, feeding KO mice HFD did not exacerbate these changes, similar to TLR4, probably due to the inflammation observed in PPARβ*/*δ^-/-^ mice that reached so high levels that it can be increased by chronic low-intensity intervention such as diet.

Interestingly, our results showed a significant increase in PTP1B protein levels in the hippocampus caused by the genotype. This increase was similar to that observed after HFD intake. An analogous trend was observed for NFKβ and astrogliosis. The PTP1B expression is highly increased in activated microglia, which in turn is enhanced due to pro-inflammatory processes, suggesting that it is an important positive regulator of inflammation (Song et al., 2016). However, PTP1B has not only been related to neuroinflammatory mechanisms. In fact, it has been demonstrated that it is a key regulator of insulin sensitivity since mice with *Ptp1b* gene deletion present a reduction of insulin resistance, turning it into a promising target not only for the design of anti-diabetic drugs (Elchebly et al., 1999; Zhang and Lee, 2003) but also to elude synaptic alteration and cognitive loss (Fuentes et al., 2012; Ricke et al., 2020), since PTP1B activity in the hippocampus has been correlated with impaired neuronal insulin signaling (Wang et al., 2017). Taking all these data into account, our results confirm the studies performed by de la Monte and colleagues that demonstrated that downregulation of PPARβ/δ could be linked to both inflammation and insulin resistance in the brain (De La Monte and Wands, 2006).

It is well known that IR and insulin signaling play an important role in neuronal physiological functions, contributing to synapse formation, neuronal plasticity (Kleinridders et al., 2014; Agostinone et al., 2018), and reduction in neuroinflammatory process, which all together promote the cognitive function (Chiu et al., 2008; Craft et al., 2012; Carlson et al., 2014). Once IR is activated, AKT is phosphorylated/activated, which in turn phosphorylates various biological substrates, including GSK3β. In this context, the dysfunction of this pathway occurs, which has been associated with insulin resistance leading to memory impairment (Qi et al., 2015; Zhang et al., 2018b; Yang et al., 2020). In our study, p-AKT and p-GSK3β protein levels showed a significant decrease in the hippocampus of PPARβ/δ^-/-^ mice in agreement with spine dendritic reduction and increased neuroinflammation observed in this genotype. Moreover, we did not observe changes in the diet variable, probably due to the fact that the lack of this receptor interferes in the attachment of fat to its receptor. Of note, an increase in IR protein level was observed in these mice, suggesting that it could be a compensatory mechanism to deal with this insulin signaling disruption. These results concur with previous studies performed by Buck and colleagues who demonstrated that when insulin-like growth factor 1 receptor (a co-receptor of insulin signaling pathway) was inhibited, a compensatory IR activation was observed (Buck et al., 2010), demonstrating the key role of PPARβ/δ in this process.

## Conclusion

**FIGURE 8:**
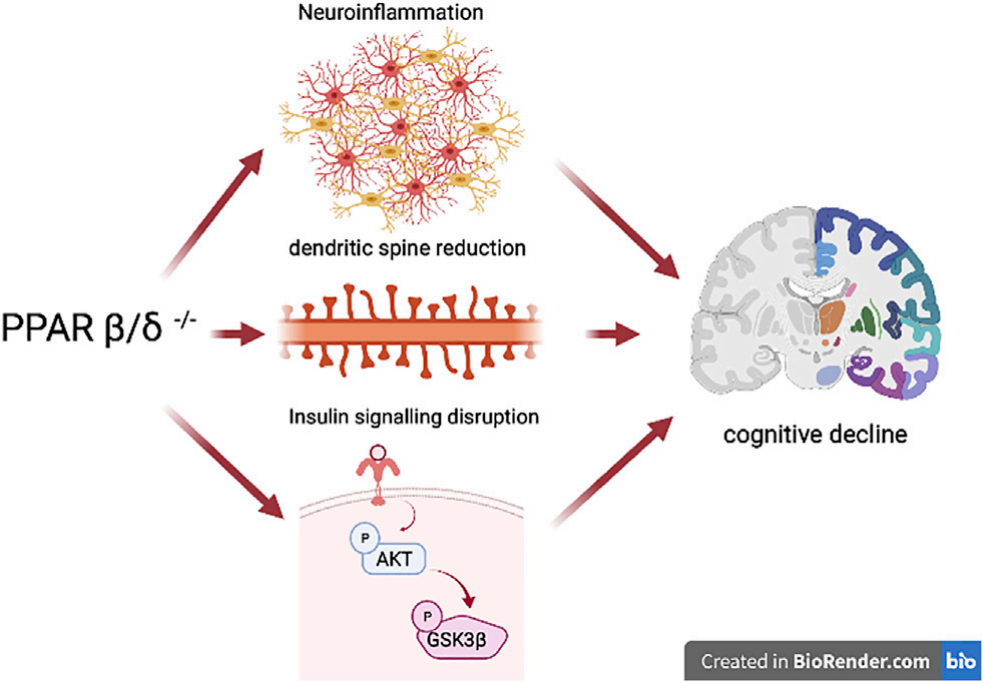
Schematic representation of the effects of PPARβ/δ deficiency. The figure shows how this receptor is a key molecule in the development of some of the most important features of memory impairment such as neuroinflammation, reduction in the number of dendritic spines, as well as an alteration of synaptic biomarkers, and insulin signaling disruption. In this way, this transcription factor represents a promising target for the treatment and improvement of memory impairment, an important hallmark of neurodegenerative diseases.

In conclusion, the present study demonstrates for the first time that PPARβ/δ deficiency in the brain constitutes not only a new risk factor associated with cognitive loss in neurological diseases but also a key molecule targeting the pivotal pathways leading to memory impairment which include neuroinflammation, insulin resistance, dendritic spine regulation, and synaptic plasticity, among others (Figure 8). On the other hand, we observed that HFD intake affects mechanisms involved in the memory process in the presence of the receptor, but did not exacerbate this process in the presence of PPARβ/δ. Therefore, PPARβ/δ provides a new and promising therapeutic target in order to design novel strategies focused on curbing or improving memory impairment present in most neurological diseases.

## Data Availability

The original contributions presented in the study are included in the article/Supplementary Material. Further inquiries can be directed to the corresponding author.

## Abbreviations

AD, Alzheimer’s disease; AKT/p-AKT, protein kinase B/phospho-protein kinase B; BDNF, brain-derived neurotrophic factor, CNS, central nervous system; CT, conventional chow; DBN1, drebrin 1; DG, dentate gyrus; GAPDH, glyceraldehyde-3-phosphate dehydrogenase; GFAP, glial fibrillary acidic protein; GSK3β/p-GSK3β, glycogen synthase kinase 3 beta; GTT, glucose tolerance test; HFD, high-fat diet; IBA1, ionized calcium-binding adapter molecule 1; IR, insulin receptor; ITT, insulin tolerance test; KO, knock-out; MWM, Morris Water Maze; NFKβ, nuclear factor kappa B; NORT, novel object recognition test; NSAIDs, nonsteroidal anti-inflammatory drugs; PB, phosphate buffer; PFA, paraformaldehyde; PPARs, peroxisome proliferator-activated receptors; PSD95, density protein 95; PTP1B, protein tyrosine phosphatase; T2DM, type 2 Diabetes Mellitus; TLR4, Toll-like receptor 4; WT, wild type.
