# Supplementary figures and images for: Peroxisomal Proliferator-Activated Receptor β/δ Deficiency Induces Cognitive Alterations
Source: Front Pharmacol. 2022 Jul 11;13:902047. doi: 10.3389/fphar.2022.902047 (PMC9310104; doi:10.3389/fphar.2022.902047)

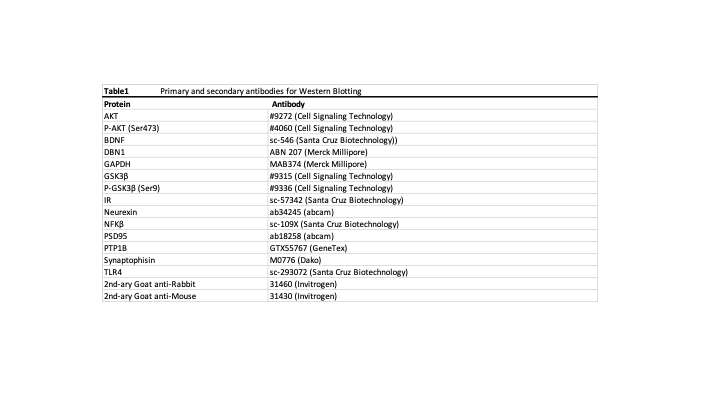

Supplement: Supplementary file 1 [file Image1.TIFF]

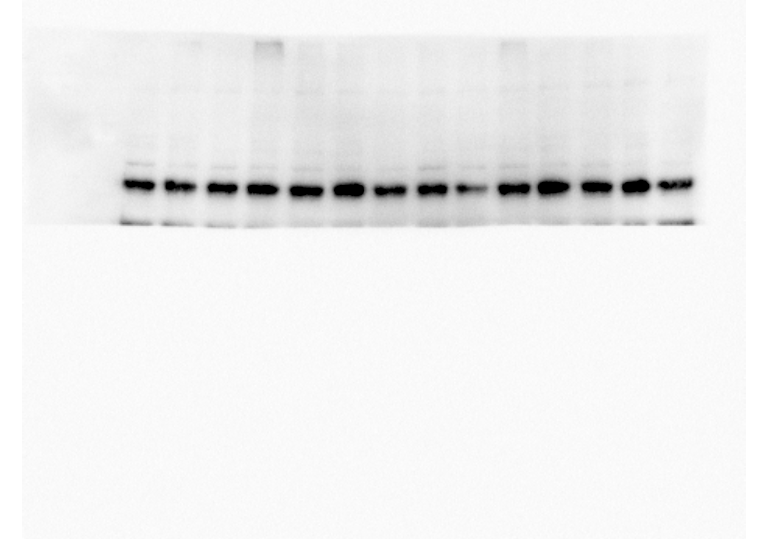

Supplement: Supplementary file 2 [file DataSheet1.ZIP › PAPER PPAR/AKT/AKT.tif]

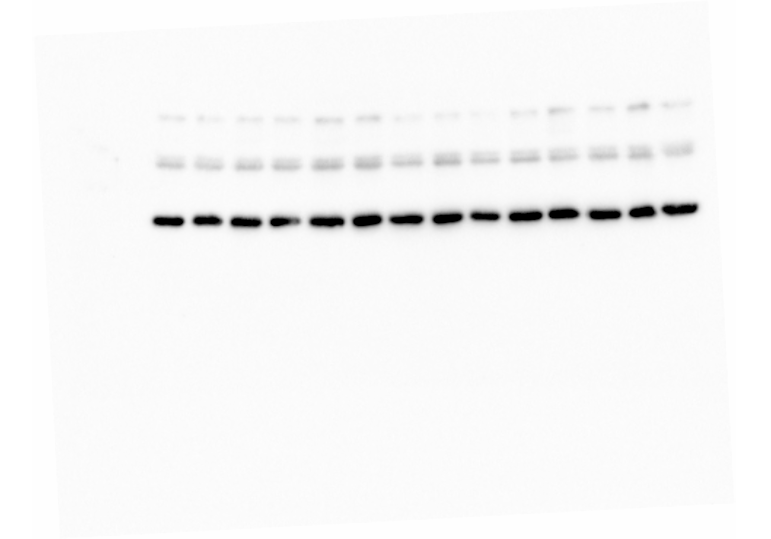

Supplement: Supplementary file 2 [file DataSheet1.ZIP › PAPER PPAR/AKT/GAPDH AKT.tif]

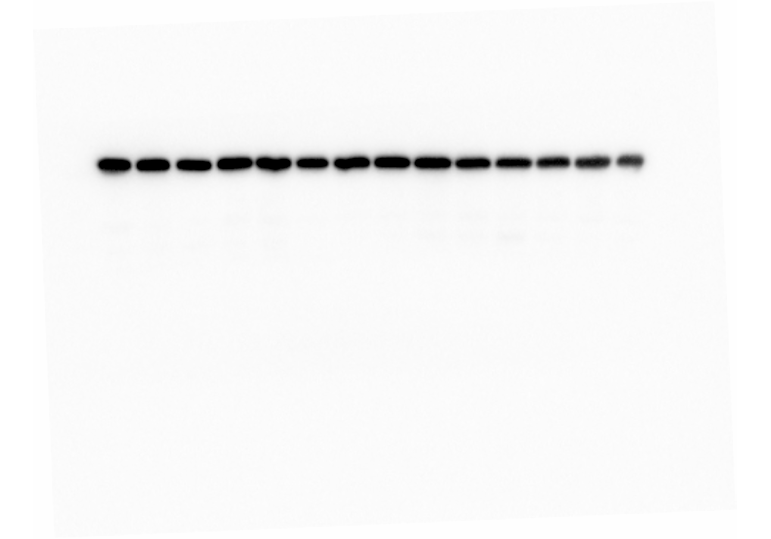

Supplement: Supplementary file 2 [file DataSheet1.ZIP › PAPER PPAR/BDNF/BDNF.tif]

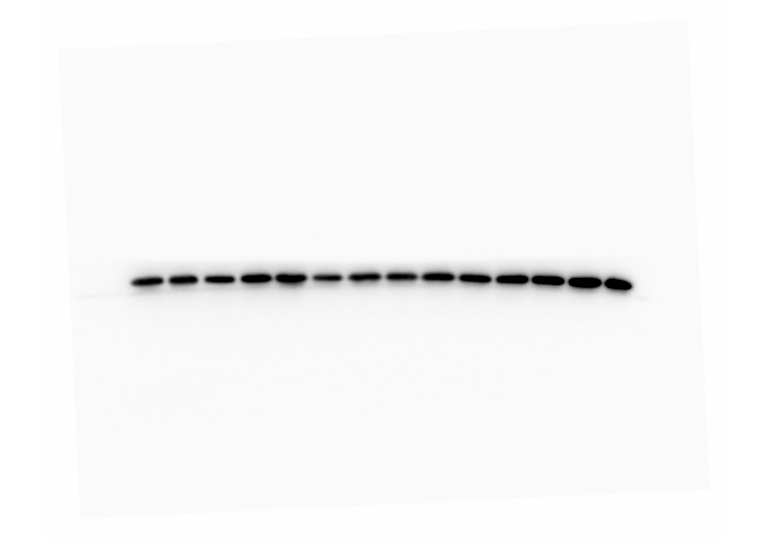

Supplement: Supplementary file 2 [file DataSheet1.ZIP › PAPER PPAR/BDNF/GAPDH BDNF.tif]

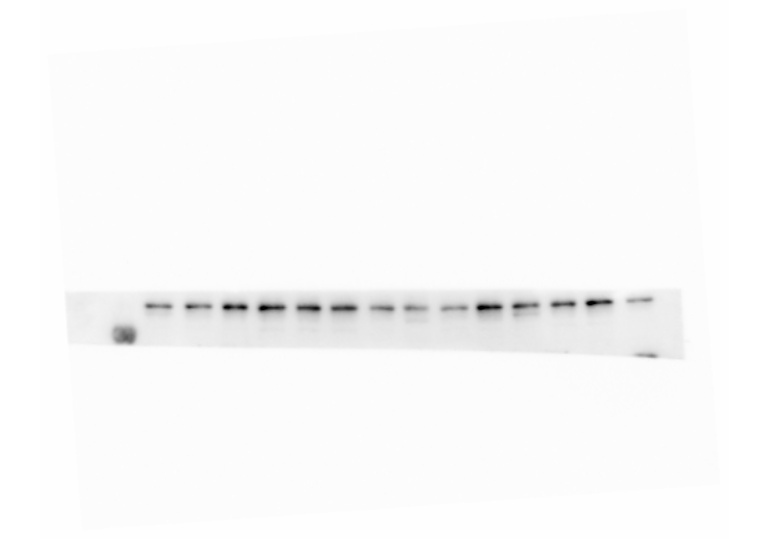

Supplement: Supplementary file 2 [file DataSheet1.ZIP › PAPER PPAR/DBN1/DBN1.tif]

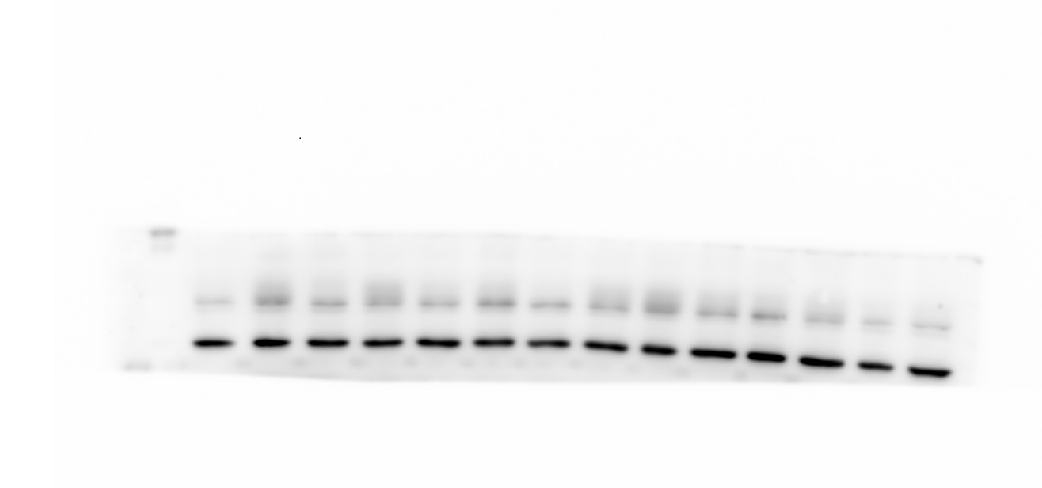

Supplement: Supplementary file 2 [file DataSheet1.ZIP › PAPER PPAR/DBN1/GAPDH DBN1.tif]

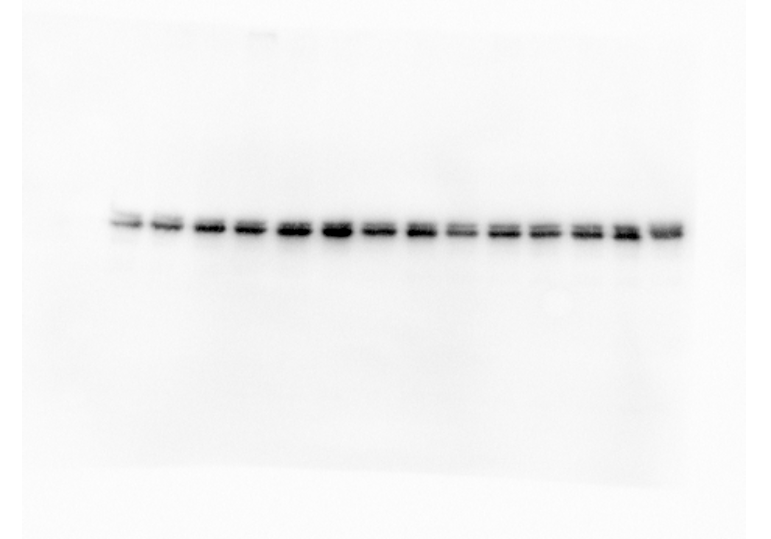

Supplement: Supplementary file 2 [file DataSheet1.ZIP › PAPER PPAR/GSK3B/GSK3B.tif]

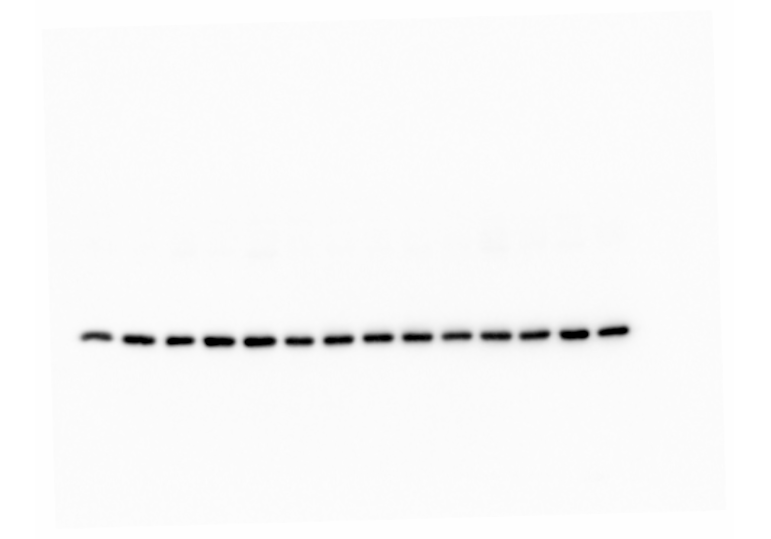

Supplement: Supplementary file 2 [file DataSheet1.ZIP › PAPER PPAR/IR/GAPDH IR.tif]

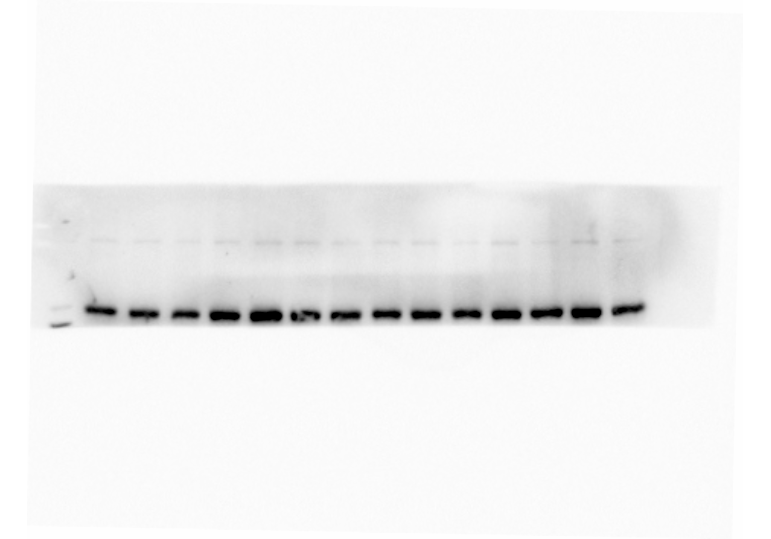

Supplement: Supplementary file 2 [file DataSheet1.ZIP › PAPER PPAR/IR/IR.tif]

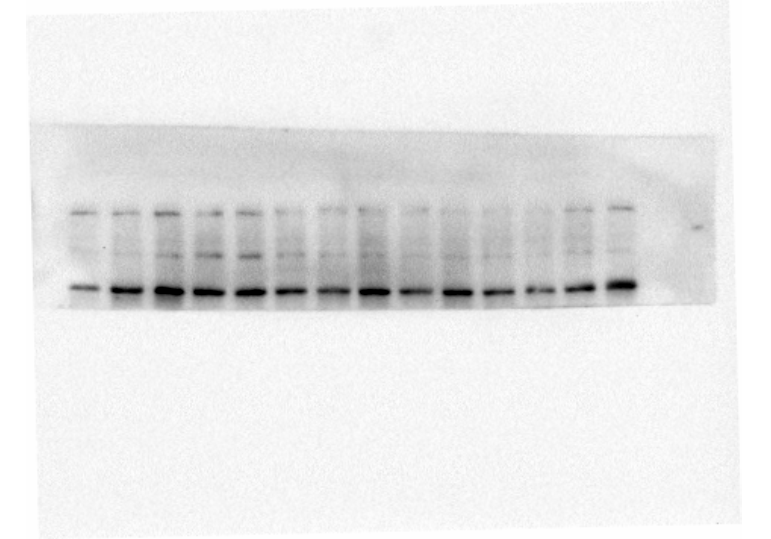

Supplement: Supplementary file 2 [file DataSheet1.ZIP › PAPER PPAR/Neurexin/Neurexin.tif]

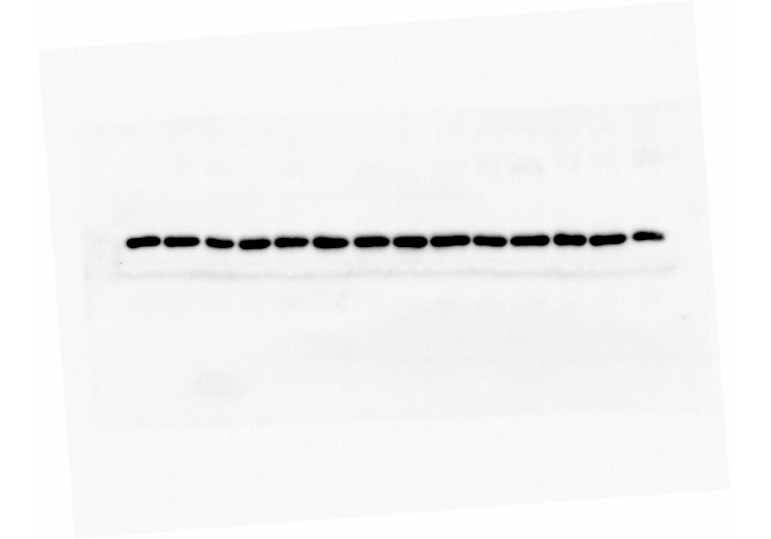

Supplement: Supplementary file 2 [file DataSheet1.ZIP › PAPER PPAR/NFKB/GAPDH NFKB.tif]

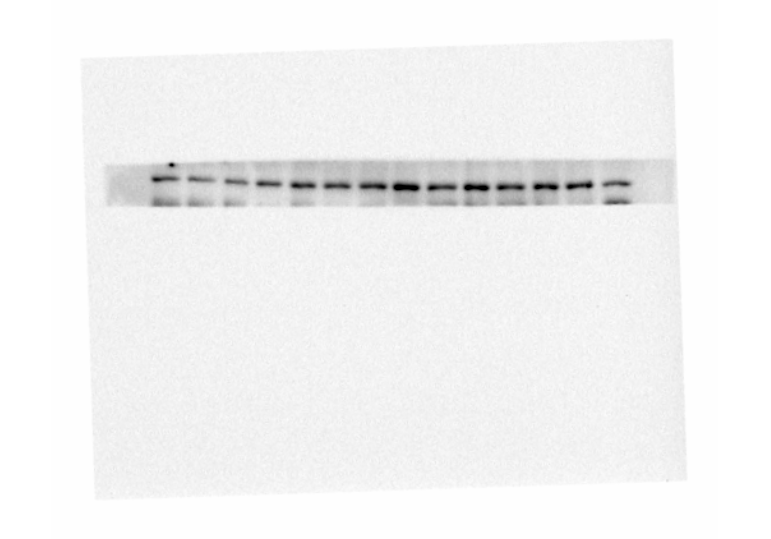

Supplement: Supplementary file 2 [file DataSheet1.ZIP › PAPER PPAR/NFKB/NFKB.tif]

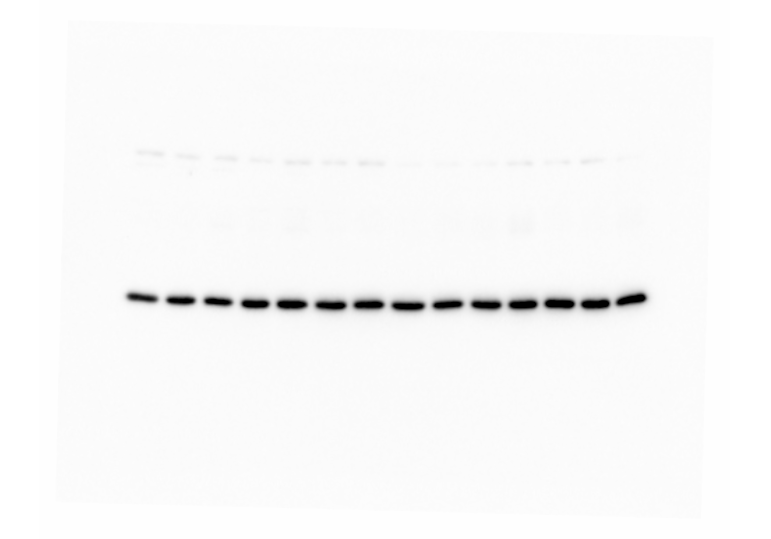

Supplement: Supplementary file 2 [file DataSheet1.ZIP › PAPER PPAR/P-AKT/GAPDH P-AKT.tif]

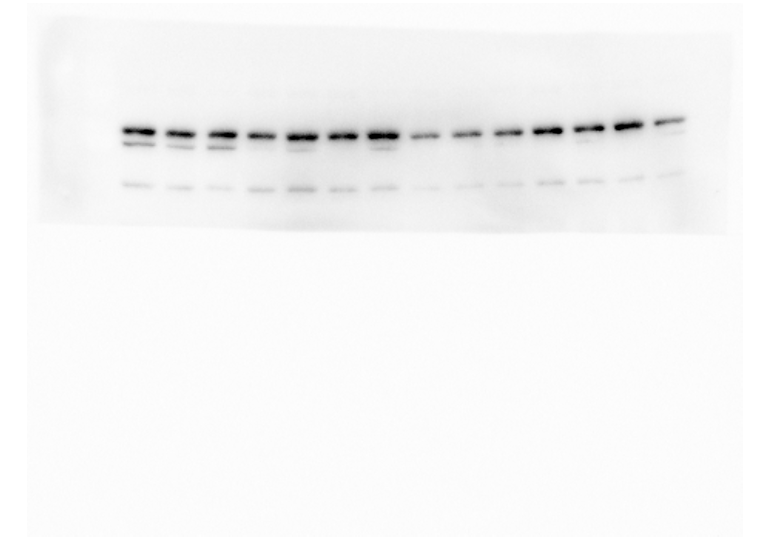

Supplement: Supplementary file 2 [file DataSheet1.ZIP › PAPER PPAR/P-AKT/P-AKT.tif]

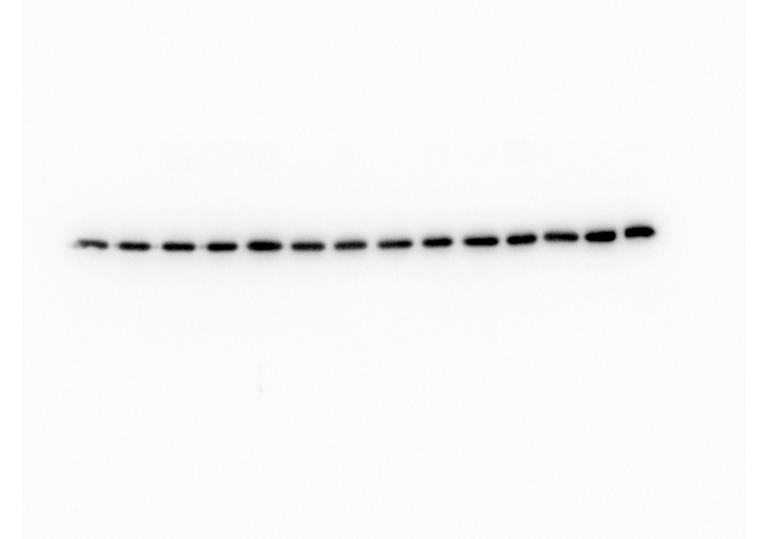

Supplement: Supplementary file 2 [file DataSheet1.ZIP › PAPER PPAR/P-GSK3B/GAPDH P-GSK3B.tif]

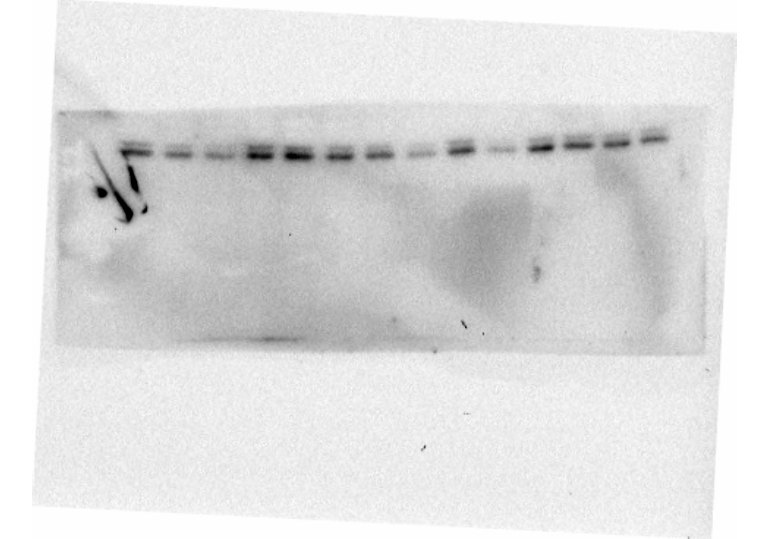

Supplement: Supplementary file 2 [file DataSheet1.ZIP › PAPER PPAR/P-GSK3B/P-GSK3B.tif]

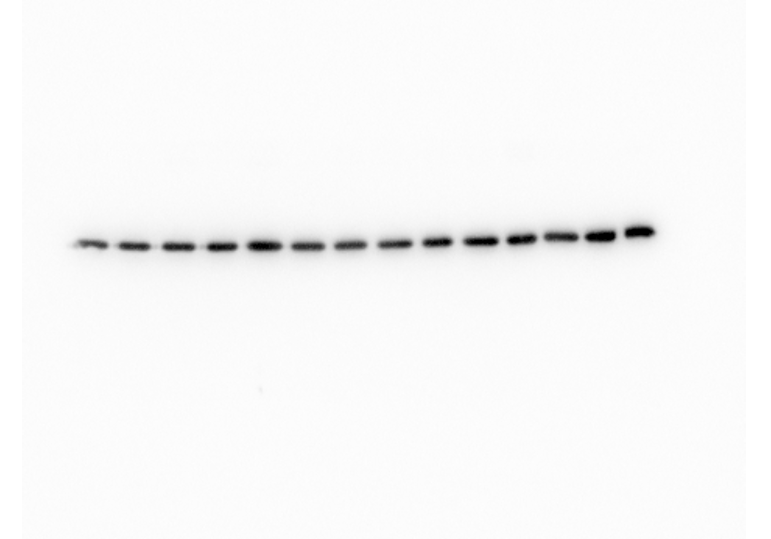

Supplement: Supplementary file 2 [file DataSheet1.ZIP › PAPER PPAR/PSD95/GAPDH PSD95.tif]

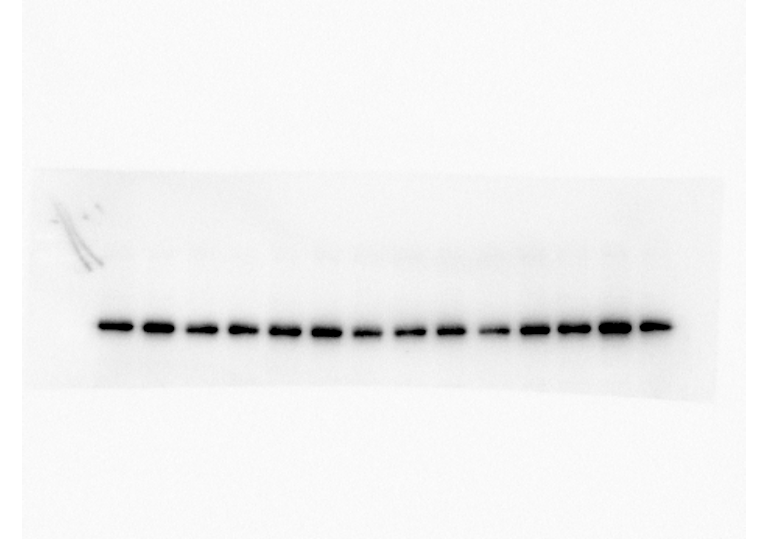

Supplement: Supplementary file 2 [file DataSheet1.ZIP › PAPER PPAR/PSD95/PSD95.tif]

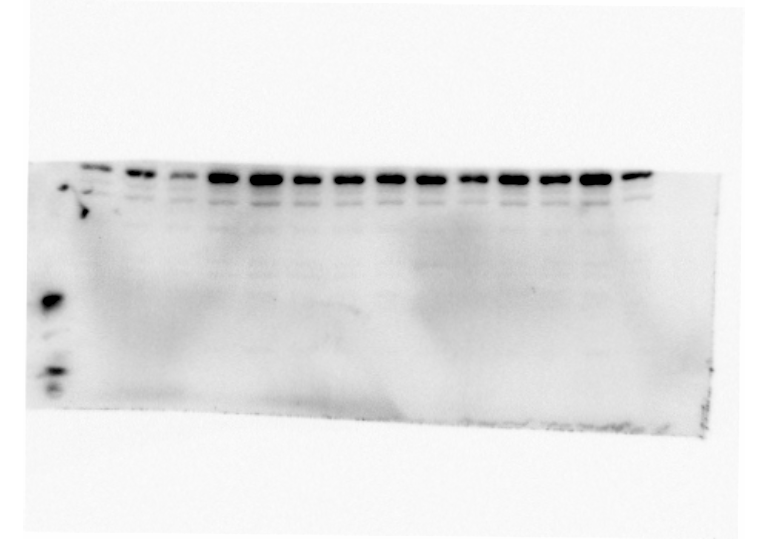

Supplement: Supplementary file 2 [file DataSheet1.ZIP › PAPER PPAR/PTP1B/PTP1B.tif]

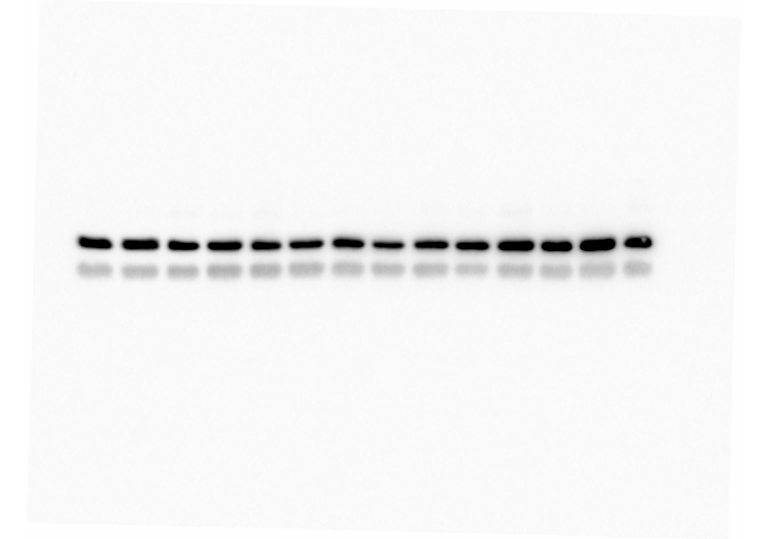

Supplement: Supplementary file 2 [file DataSheet1.ZIP › PAPER PPAR/Synaptophisin/Actin Synaptophisin.tif]

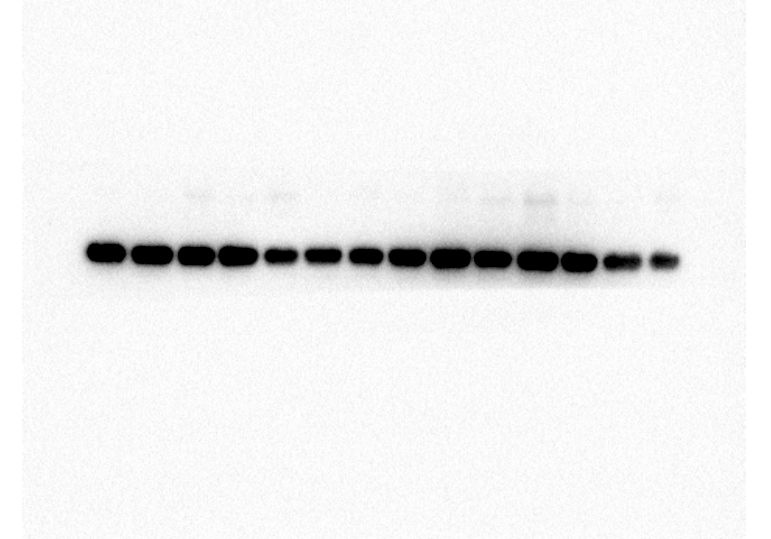

Supplement: Supplementary file 2 [file DataSheet1.ZIP › PAPER PPAR/Synaptophisin/Synaptophisin.tif]

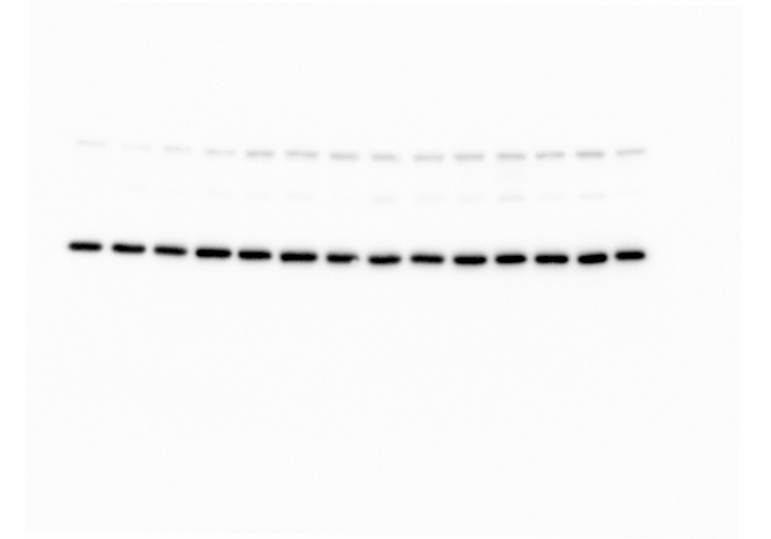

Supplement: Supplementary file 2 [file DataSheet1.ZIP › PAPER PPAR/TLR4/GAPDH TLR4.tif]

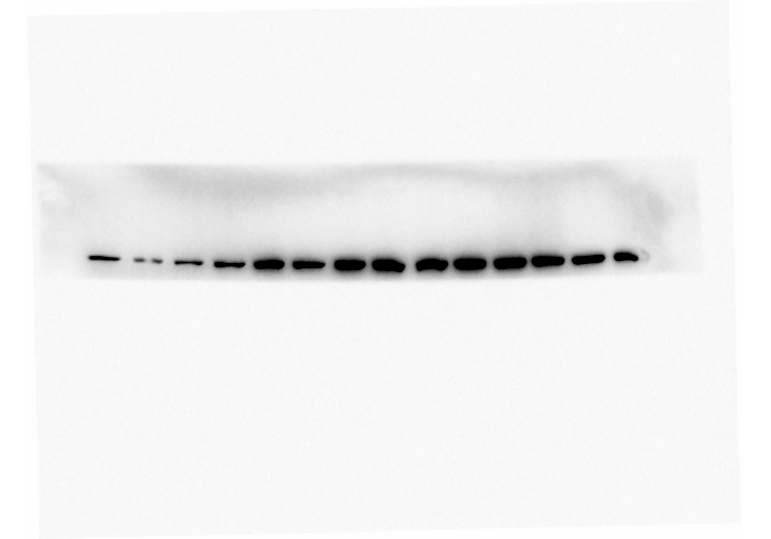

Supplement: Supplementary file 2 [file DataSheet1.ZIP › PAPER PPAR/TLR4/TLR4.tif]

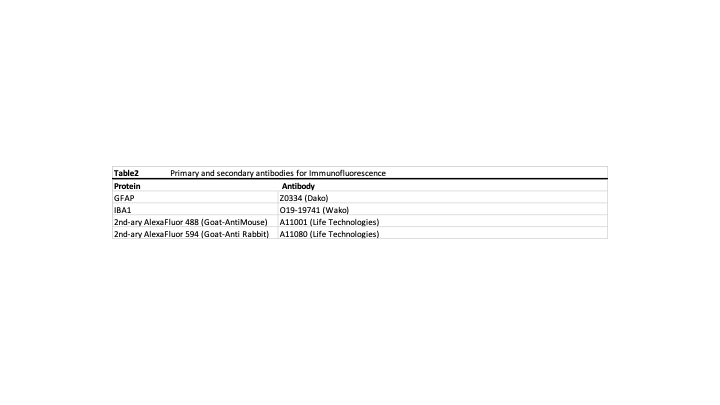

Supplement: Supplementary file 3 [file Image2.TIFF]
